# Real-time pneumonia prediction using pipelined spark and high-performance computing

**DOI:** 10.7717/peerj-cs.1258

**Published:** 2023-03-09

**Authors:** Aswathy Ravikumar, Harini Sriraman

**Affiliations:** School of Computer Science and Engineering, Vellore Institute of Technology, Chennai, Tamil Nadu, India

**Keywords:** Parameter server, Convolutional neural network, Spark, Data parallel model, Prediction model, Pneumonia, Distributed deep learning, High performance computing

## Abstract

**Background:**

Pneumonia is a respiratory disease caused by bacteria; it affects many people, particularly in impoverished countries where pollution, unclean living standards, overpopulation, and insufficient medical infrastructures are prevalent. To guarantee curative therapy and boost survival chances, it is vital to detect pneumonia soon enough. Imaging using chest X-rays is the most common way of detecting pneumonia. However, analyzing chest X-rays is a complex process vulnerable to subjective variation. Moreover, the data available is growing exponentially, and it will take hours and days to train the model to predict pneumonia. Timely prediction is significant to guarantee a better cure and treatment. Existing work provided by different authors needs more precision, and the computation time for predicting pneumonia is also much longer. Therefore, there is a requirement for early forecasting. Using X-ray picture samples, the system must have a continuous and unsupervised learning system for early diagnosis.

**Methods:**

In this article, the training time of the model is accelerated using the distributed data-parallel approach and the computational power of high-performance computing devices. This research aims to diagnose pneumonia using X-ray pictures with more precision, greater speed, and fewer processing resources. Distributed deep learning techniques are gaining popularity owing to the rising need for computational resources for deep learning models with several parameters. In contrast to conventional training methods, data-parallel training enables several compute nodes to train massive deep-learning models to improve training efficiency concurrently. Deploying the model in Spark solves the scalability and acceleration. Spark’s distributed processing capability reads data from multiple nodes, and the results demonstrate that training time can be drastically reduced by utilizing these techniques, which is a significant necessity when dealing with large datasets.

**Results:**

The proposed model makes the prediction 1.5 times faster than the traditional CNN model used for pneumonia prediction. The model also achieved an accuracy of 98.72%. The speed-up varying from 1.2 to 1.5 was obtained in the synchronous and asynchronous parallel model. The speed-up is reduced in the parallel asynchronous model due to the presence of straggler nodes.

## Introduction

All organizations, from tech titans to startups, store user data and, in some circumstances, purchase data from other businesses. Every business and industry stores data for many goals, including future research, marketing, and consumer manipulation. Nonetheless, this massive volume of data is meaningless unless we create the required tools to extract relevant information from it. According to experts, the quantity of data will continue to rise exponentially shortly. Implementing different Deep Learning algorithms on massive data may be easier with Big Data tools, especially when dealing with very complicated medical datasets. To satisfy this demand, we use an accelerated discovery of pneumonia, and following administration of the appropriate treatment may aid significantly in preventing patients’ conditions from deteriorating, which may eventually result in mortality (Alharbi & Hosni Mahmoud, 2022). Several technologies, such as genetics and imaging, have emerged in recent decades to provide detailed healthcare information. Chest X-ray images are the preferred method for diagnosing pneumonia; nevertheless, these visuals are not always clear and are occasionally misclassified as benign irregularities or even other illnesses by specialist practitioners, resulting in the administration of the incorrect medication and a subsequent worsening of the medical illness. Therefore, it is necessary to develop an intelligent and automated model to aid physicians with the diagnosis of different varieties of pneumonia using chest X-ray data.

Deep learning, sometimes called deep structured learning, is a subset of sophisticated machine learning methods. It is built on algorithms that use mathematical operations and are inspired mainly by artificial neural networks (Aydin & Guldamlasioglu, 2017). The dramatic improvement in prediction performance in the past century was aided partly by developments in neural network training technologies, which have enabled the training of bigger models on more enormous datasets than ever. While modern GPUs and custom accelerators have significantly accelerated the training of neural networks, training development progresses to hamper the predictive efficiency and application breadth of these technologies. Accelerating neural network training techniques has the potential to have a substantial influence on a broad range of essential application areas. Faster training may result in considerable improvements in model quality by allowing professionals to learn more data (Anil et al., 2020) and reducing the time required for iterations, allowing researchers to explore novel ideas and setups more rapidly. Quick training also enables the deployment of neural networks in situations where models must be updated frequently, such as when new training data is added or discarded (Jin et al., 2022). In deep learning, CNN (convolutional neural network) collects features from pictures and manages the complete feature engineering process. Data parallel models are needed to predict diseases and illnesses since the data is growing exponentially and requires timely faster results (Das, Roy & Mishra, 2022). Not only has the volume of data exploded in recent years, but the nature and format of that information have also shifted dramatically. Most of this necessitates the establishment of an effective and beneficial platform for Big Data processing. Large-scale massive data collection is described as data of a terabyte or more in size that cannot be processed or stored using conventional computer methods (Baby & Ravikumar, 2014; Woolf, 2017). As deep learning has grown in popularity, numerous architectures for integrating multi-core GPU systems into distributed systems have emerged, including Yahoo’s TensorFlow on Spark, Databricks’ Deep Learning Pipeline for Apache Spark, Intel’s BigDL/Analytics Zoo, Skymind’s DL4J, and Elephas’ Distributed Deep Learning with Keras & Spark. Hadoop and Spark are generally used to analyze and forecast large amounts of data.

Spark excels at converting large datasets and applying built-in machine learning algorithms through Spark MLlib; it does not enable the creation of bespoke algorithms using deep learning frameworks such as Google’s TensorFlow and, in particular, its handy Keras (https://keras.io/) API. Elephas was the first open-source framework to provide Keras-on-Spark distributed training. Later, libraries like Yahoo’s TensorFlow on Spark, which does not adhere to the Keras API design guidelines, were developed. Other popular distributed deep learning frameworks have arisen in recent years, such as the robust Horovod (Sergeev & Del Balso, 2018), which previously lacked Spark support. BigDL (Dai et al., 2019) is another framework worth highlighting, mainly when used with Intel’s Analytics Zoo. Elephas is tightly coupled to several of Spark’s essential abstraction layers. Besides integrating Spark’s resilient distributed datasets (RDDs), Elephas supports MLlib models, Spark machine learning estimators, ensemble modeling, and distributed inference.

A DL model that has been trained with more extensive and varied data would be more reliable and resilient. However, in some real-world applications, such as healthcare applications, the data gathered by a single hospital is often restricted, and the bulk of enormous and diversified data is frequently divided among numerous companies. As a result, it drives researchers to perform DL in a distributed manner, in which the data user would want to construct DL models utilizing data dispersed among several data owners (Han et al., 2022). Nevertheless, the data providers would only be cautious and willing to engage in the data user’s distributed deep learning if the data user’s protocol addresses the data owners’ significant worries over protecting their data. It has been shown, for instance, that private information may be inferred during the learning experience (Nasr, Shokri & Houmansadr, 2019) and that the membership of training data can be determined using the trained model (Ali et al., 2022). Therefore, it is essential to create an efficient distributed deep learning method that protects the privacy of medical data analysis in real-time.

This research aims to diagnose pneumonia using X-ray pictures with more precision, incredible speed, and fewer processing resources. The associated publications concentrating on diagnosing pneumonia using X-rays employed models with several convolutional and great depths. A few works attained perfect precision, but their calculations demand a great deal of processing power and time. The primary contribution made by this study is the development of accelerated deep learning for identifying pneumonia utilizing chest X-ray pictures with balanced effectiveness in terms of reliability and scalability and the provision of a low-cost tool for healthcare and radiology professionals. The following goals have been met:

- Using the CNN algorithm to diagnose pneumonia using chest X-ray pictures as a feature extraction and classification method.
- Acceleration of the model using the master-slave data parallel model in Spark.
- Acceleration using both synchronous and asynchronous models in the parameter server.
- Utilizing the computational power of HPC.

The manuscript is organized as an introduction in which the need for the study, the background, and the main objectives are highlighted. The following section gives an overview of the related works in Pneumonia detection using X-ray images. Next, the materials and method section is given, which explains the dataset, the proposed data-parallel model, and the main modules in the model. The following section gives the implementation details of the proposed method, experimental environment, and algorithms. The result analysis and the comparison with the existing models are given in the next section. Following it, the principal findings and discussions are given, and the last section gives the conclusion and future scope.

## Related Works

Mishra, Kang & Woo (2020) showed a descriptive and predictive analysis of Big Data on Cloud Computing by constructing recommendation models utilizing a conventional method. Using typical sequential classification algorithms, Patel (2017) assessed Amazon product reviews as excellent or negative. In Carneiro et al. (2018) used the Spark platform only for descriptive analysis. For neural network training, the trend in hardware development is toward higher data parallelism. Specialized systems based on GPUs or custom ASICs coupled with high-performance connection technologies provide unprecedented data parallelism with undetermined costs and benefits. If data parallelism can dramatically speed calculations beyond the limits of current systems, we should construct much more extensive systems. According to research conducted by OpenAI, the number of operations required for AI systems, which are currently measured in petaflops, has been growing exponentially and doubling every 3.4 months since 2012. This surpasses the computing increases that single computers can achieve, even under the most optimistic interpretation of Moore’s law. There is an obvious need for systems that scale computing clusters. In Das, Roy & Mishra (2022), parallel CNN with stacked features was used for foot ulcer classification, but in this work, only the three convolutional block is parallelized. The dataset is fed into a single node; multiple nodes are not used for classification. In (Guan et al., 2022a, 2022b; Guan & Loew, 2017), the parallel attention augmented block is introduced in the CNN model for a more effective prediction of Alzheimer’s disease. This work also concerns improving the CNN model rather than the time taken for the model training or computational efficiency.

In Carneiro et al. (2018) evaluated the performance of deep learning applications on Google Colab, Distributed Hardware, and Mainstream Workstations, finding that Colab’s performance was equivalent to that of specialist hardware. Gupta & Addala (2019) studied two serverless variations, one with a mapper and the other with both mappers and reducers. They observed that the execution time of the machine is sublinear concerning the number of invoked reducers. Combining Jupyter notebooks with Spark (Tianshi & Wei, 2016) created a scalable large-scale data mining framework. Also, using Dockers to create a scalable environment for data processing operations (Martín-Santana et al., 2019). The authors also successfully implemented long-short term memory networks using Spark. Zhang, Choromanska & LeCun (2015), Chen et al. (2022) implemented LSTM on a cluster of nine workstations and discovered that Cluster-Based LSTM performed better than traditional LSTM in terms of root mean square error. In Aydin & Guldamlasioglu (2017) constructed LSTM on Spark using the distributed computing frameworks Keras and Elephas, and the resulting model was deemed reliable. Using a cluster of seven computers to deploy LSTM and GRU into three hidden layers for energy load forecasting, Kumar et al. (2018) discovered that GRU outperformed LSTM. Additionally, it was revealed that clusters minimize training time by a factor of six. A detailed analysis of the implementation of the CNN model in TPU and GPU is done on different benchmark applications (Ravikumar et al., 2022). The hardware accelerators efficiently process many machine learning algorithms in a single-node, multimode, and cloud environments (Harini & Ravikumar, 2021; Harini, Ravikumar & Garg, 2021; Harini, Ravikumar & Keshwani, 2022).

Medical segmentation and classifications are widely implemented in deep learning models (Robin, John & Ravikumar, 2021; John, Ravikumar & Abraham, 2021). Many academics have utilized deep learning to accurately diagnose lung infections and illnesses using chest X-rays throughout the last decade. Stephen et al. (2019) built a CNN approach from scratch to gather characteristics from chest X-ray pictures to achieve high classifier performance and utilized it to determine how likely a patient has pneumonia, unlike earlier research that relied on manual features.

Recently, the TL approach has gained much popularity, primarily because it makes CNN models more efficient, less expensive, and less dependent on inputs. In addition, they were able to categorize chest X-ray pictures accurately. According to the findings of this study, pneumonia can be identified using deep CNNs. They employ systematic techniques as a component of our data categorization strategy to minimize computing expenses Cheplygina, de Bruijne & Pluim (2019). Transfer learning methods on ImageNet with four pre-trained CNN architectures were employed to identify pneumonia. To categorize chest radiography pictures, they used three distinct classification methods. Several recently published works address this issue by attempting to identify pneumonia using deep CNN algorithms with less convolutional layers, as it is in Chen et al. (2022), Mahmoudi et al. (2022). To understand CNN architecture, they employed a region of interest that only contained the lungs instead of the complete picture. However, these methods still need to be improved in identifying pneumonia with a high degree of accuracy. Zhang et al. (2021), the authors recognized pneumonia symptoms with an efficiency greater than 96% using transfer learning models.

## Materials and Methods

**Figure 1:**
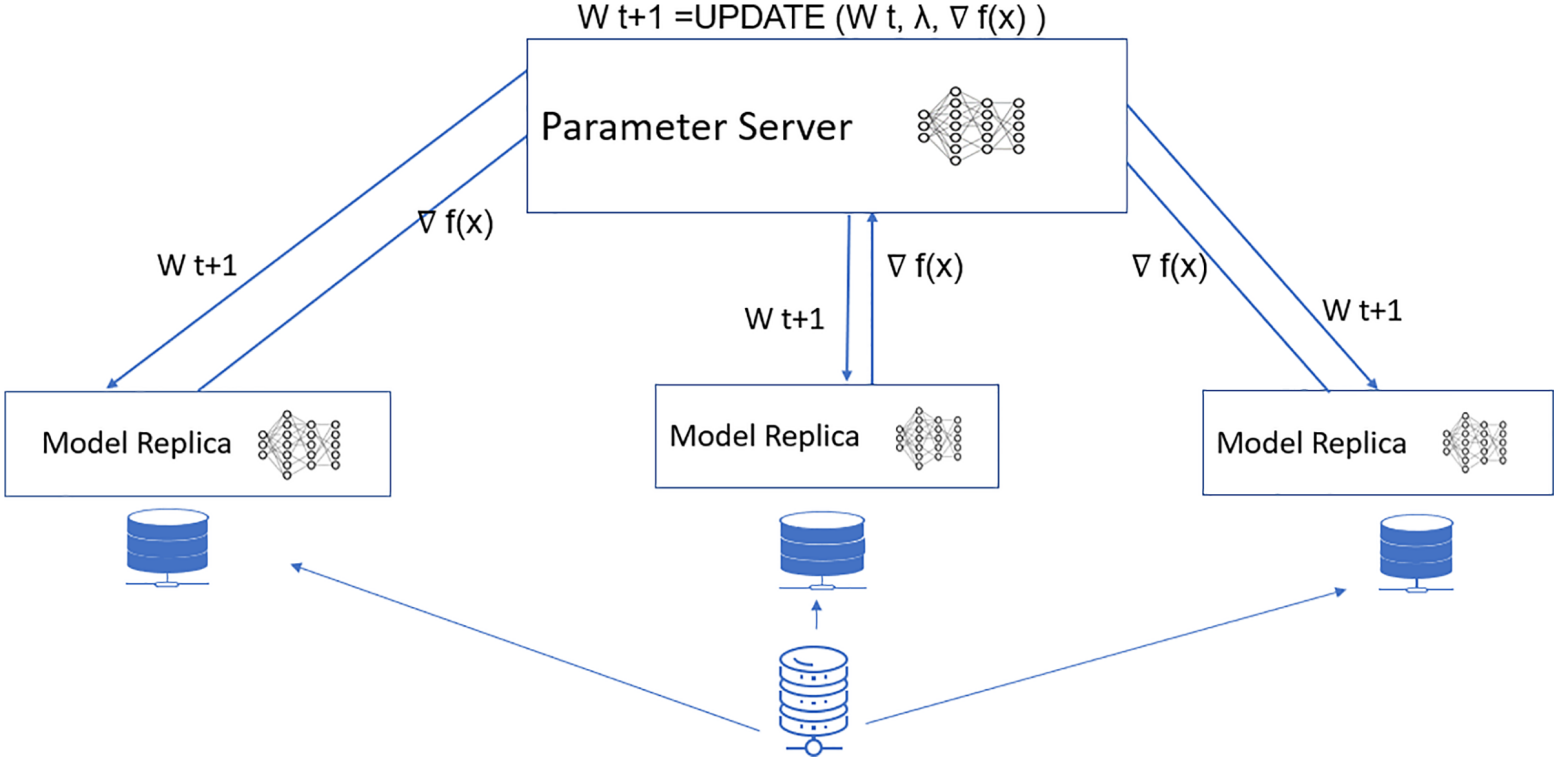
Proposed data parallel parameter server model.

**Figure 2:**
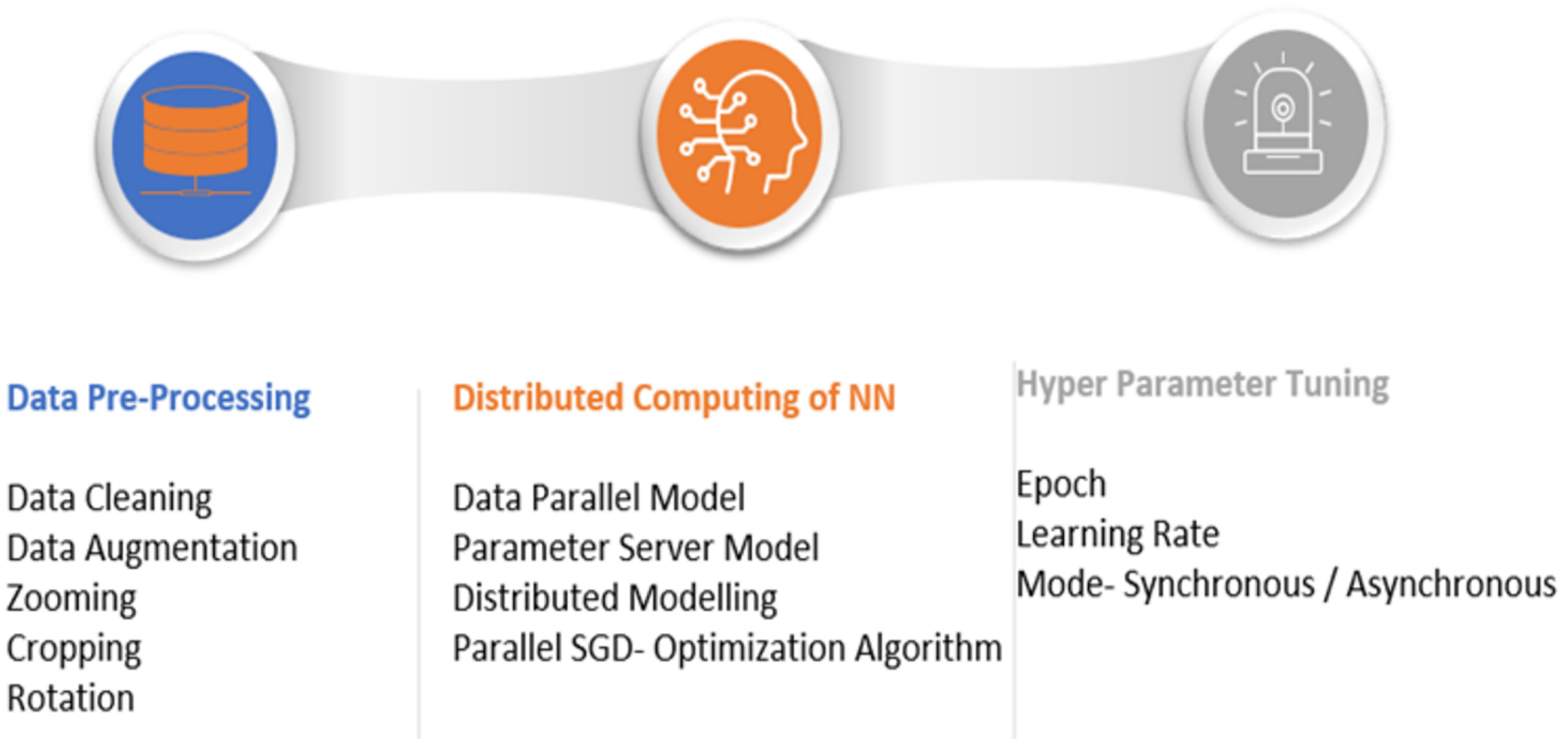
Steps in proposed model.

The proposed model mainly involves the data parallel parameter server model with a parallel stochastic gradient descent algorithm for the neural network update. The proposed model is shown in Fig. 1. The significant steps involve the data preprocessing, distributed computing model, and fine-tuning of the neural network hyperparameters, as shown in Fig. 2. This section begins with a discussion of the datasets used for the research, followed by a description of the proposed pneumonia detection technique.

Figure 1 shows the proposed model for accelerated pneumonia prediction. In this model, the data is distributed among the worker nodes, and the model replica is taken at each node. There is a central parameter server model for the model synchronization. The weight update and synchronization take at the parameter server nodes. The Wt+1 represents the weight for the next iteration of the deep learning model. It is updated in the parameter server model with the W(weights) received from each worker node, $\nabla f\left( x \right)$ represents the optimization function and λ is the learning rate.

### Dataset

The dataset (“Chest X-Ray Images (Pneumonia)”, Mooney, 2018) contains 5,863 X-ray images with class labels ‘Pneumonia’ and ‘Normal’. Chest X-ray pictures (anterior-posterior) were chosen retrospectively from pediatric patients aged 1 to 5 years at Guangzhou Women and Children’s Medical Center in Guangzhou. This study focuses on bacterial pneumonia. Bacterial pneumonia is a lung infection that is caused by specific bacteria. Streptococcus is the most prevalent cause. However, other bacteria can also be responsible. At a younger age and in generally good condition, these bacteria can exist in the esophagus without creating any problems. However, if the body’s defenses are compromised for whatever reason, the germs can enter the lungs. When this occurs, the air sacs of the lungs become infected and swollen. As a result, they fill with fluid, which leads to pneumonia.

Before training, data preparation is often done to filter, cleanse, and enrich the dataset. Since the pneumonia dataset has already been purged by removing duplicates and low-quality photos, we merely use data augmentation at this step. Data augmentation is the process of creating new data from current data *via* the use of specific techniques. Popular data enhancement techniques include scaling, horizontal and vertical picture flipping, zooming, cropping, and image rotation. Since this work aims to diagnose X-ray pictures, cropping, vertical flip, and magnification are inappropriate. Therefore, we solely employ scaling, horizontal flipping, and rotation.

### Data parallelism

Data parallelism is a straightforward and extensively used approach for expediting the training of neural networks. Parallelism in data processing refers to distributing training instances over many processors to compute gradient modifications and then aggregating these locally calculated updates. Data parallelism is model-independent and applicable to any neural network design that decomposes the training objective into a sum over training examples. By contrast, the maximum level of model parallelism (distribution of attributes and processing over many processors for much the same training examples) is model-dependent. While data parallelism may be easier to construct, large-scale systems should incorporate all available parallelism. In parallel data techniques, each executor receives a copy of the whole model, parallelizing gradient descent processing by splitting data into smaller chunks. After receiving a gradient from each executor, a parameter server integrates the findings of each subset and synchronizes the model parameters amongst the executors. This may be accomplished synchronously or asynchronously. On the other hand, asynchronous (Anil et al., 2020) approaches outperform synchronous methods in homogenous settings where nodes share the exact hardware specifications and connect over a trustworthy network of communication. To begin, executors do not wait for others to commit before beginning the following data processing batch. Second, the asynchronous technique is more resilient to node failure. Even if one node fails, the other nodes will continue to train their data partitions and get fresh updates from the parameter server. Synchronous data parallelism is a training paradigm in which training stages are done sequentially and synchronously. During the training run time, a succession of locking mechanisms allows identification.

### Parameter server

The architecture of a parameter server consists of parameter server nodes and worker nodes (Li et al., 2013). The clients are responsible for learning the parameters based on the data they receive and updating the hyperparameters based on their learning outcomes, whereas the PS stores the changed parameters and handles synchronization. The Single Parameter Server technique is the most fundamental parameter server-based strategy, with each node functioning as a client and a single worker responsible for working and syncing the parameters. This approach employs a single node as both the PS and worker and all other nodes as worker-only nodes. In other words, just one node is employed to store and synchronize the gradient necessary for training to continue. In distributed training, there is a cluster of employees, and so far, we have seen that each worker does just one job, namely training. However, we may give each worker a distinct function, such that some serve as parameter servers and the remainder as training workers. The parameter servers are all in charge of storing the model’s parameters and updating the model’s global state. Whereas the training workers execute the actual training loop and generate gradients and losses from the data provided, the training workers conduct the training loop. The procedure is as follows:

Replicate the model across all the worker nodes, with each worker using a portion of training data.

Each worker in training retrieves parameters from parameter servers.

Each worker executes a training loop and transmits the gradients to all parameter servers, which subsequently update the model’s parameter. The main drawbacks of the PS model are:

One downside is that, at any moment, only one of the employees is utilizing the most recent version of the model, while the others use an outdated version.

If just one worker is used as a parameter server, this could create a bottleneck for large clusters and a single point of failure. Nevertheless, the bottleneck issue may be mitigated by installing numerous parallel servers.

### Parallel stochastic gradient descent algorithm

SGD is an optimization approach often used in deep learning to determine the model parameters that correspond to the best fit between expected and actual outputs. Mini-batch SGD is synchronous due to the aggregate after each run over the data, which serves as the locking mechanism. A distributed adaption of mini-batch SGD, in which each system node computes on a single mini-batch, is likewise a synchronous technique, as explained in Algorithm 1. A master node, also known as a parameter server, collects the weights that each worker calculates based on its data partition. After each run over the data, the parameter server must aggregate the weights before going to the next iteration. Some workers take longer than others; synchronization becomes a severe training barrier. Careful attention is required to reduce the likelihood of this sort of bottleneck. Moreover, this method necessitates parameter adjustments for each mini-batch iteration. In a cluster computer, this causes substantial communication costs.

**Algorithm 1:** SGD.

Initialize the learning rate λ and initial model parameters ***θ*** ­
Repeat till the end of epochs:
Select a sample from the training dataset
Initialize gradient as 0
For i = 1 to m
Calculate the gradient using equation g = g + ∇θ Loss f(x,y)
At the end of the loop i
Update model parameter using equation θ = θ – λ g, with the new gradient calculated
End For loop

The SGD algorithm is given in Algorithm 1, where the model parameters ð and learning rate λ are initialized in the forward pass of the neural network model. Then, the steps are repeated until the iterations are specified, and initially, the gradient value is set to zero. Then, it is calculated during the backpropagation of the neural network to calculate the loss. Based on the loss calculated, the new model parameters are updated, and this process continues until the loss becomes negligible (approaches zero).

Parallel SGD is an implementation of the Data-Parallel technique. This optimizer employs two distinct computer types (or nodes): a parameter server node and a client node, and works based on Algorithm 2. In the parallel SGD, the algorithm is executed parallel in multiple nodes and the gradient is calculated in the nodes for different batch sizes finally the aggregation is performed for the calculation of updated model parameters.

**Algorithm 2:** Parallel SGD.

For each iteration, parallel execute the following steps
Perform SGD on each compute node
End For
Aggregate the gradient calculated from all nodes
Broadcast the new model parameters.

## Experimental Details

The proposed model is implemented in Elephas spark using the parameter server data parallel model using a novel CNN model using the Stochastic Gradient Descent algorithm. Google Colab-Collaboratory is Google’s platform, which provides a Jupyter notebook for machine learning and deep learning applications. In addition, Colab provides the virtual machine platforms CPU, GPU, and TPU (Google Colab, 2022). Elephas distributed deep learning is implemented by Keras and Spark using the Elephas library (Elephas, 2022).

### Spark

The Spark framework (“Overview—Spark 3.3.0 Documentation”, Apache Spark, 2022) has been created at the University of Berkeley’s AMP lab since 2009 and is presently maintained by Databricks. This approach overcomes MapReduce’s shortcomings by providing a resilient distributed datasets (RDD) abstraction that performs operations in memory. Spark transforms processes’ action sequences into efficient tasks performed on the Spark engine. Spark provides a functional programming API for manipulating distributed resilient datasets (RDDs). RDDs are objects spread over several computer nodes and may be modified concurrently. Spark Core is a computational engine that manages application scheduling, distribution, and monitoring. It comprises many computing jobs distributed among executor nodes on a compute node/cluster. Spark’s scheduler will execute the tasks across the whole cluster.

Spark reduces data loading recurrence by caching data in memory, which is critical in complicated operations. Big Data systems can store and compute vast datasets larger than Gigabytes for data engineering, analysis, and even deep learning. For example, the dataset grows from 16 Gigabytes to 200 Terabytes of data. The commercialization of digital technology led to an exponential rise in the volume and variety of data collected and processed across multiple fields. As a result, individual devices’ processing and storage capacities continue to deteriorate. Thus, information and task parallelization approaches are increasingly being used to boost the effectiveness among the most demanding applications by distributing the data and analytical workloads over a cluster of processing nodes. MapReduce, a method developed by Google, is one of the most advanced parallel processing frameworks for cloud data centers (Maitrey & Jha, 2015). MapReduce is a software middleware for distributed computing and a programming language that allows applications to be rebuilt concurrently using the map () and reduce () techniques. MapReduce has had a great deal of success with batch processing.

### Elephas

The Keras (“Keras: the Python deep learning API”, Keras, 2022) in Spark has the package Elephas (“GitHub—maxpumperla/elephas: Distributed Deep learning with Keras & Spark”, Elephas, 2022) for the distributed implementation of deep learning algorithms. In Elephas, distributed modeling using the prototyping strategy is employed. Elephas aims to maintain Keras’s simplicity and utility, enabling the rapid development of distributed models that can be executed on enormous amounts of information. Furthermore, Elephas builds a class of data-parallel techniques on top of Keras using Spark’s RDDs and data frames. Spark’s ability to parallelize data processing in a durable manner using RDDs connects well with data parallelism since a Spark job parallelizes data processing over several computers. In practice, it is straightforward: a Keras model is set up on the Spark driver and then handed to a worker in its entirety, along with a portion of the data to train on. Each worker then trains the model independently and transmits the gradients back to the driver, updating the “master model” in the data-parallel manner mentioned above. Indeed, Elephas is not limited to training but also helps with distributed data parallelism for the Keras model, distributed optimization of the Keras model’s hyperparameters, and distributed ensemble model training through hyperparameter optimization.

Model creation using Keras and Elephas follows the following steps:

- Establish a Pyspark environment
- Define and assemble the Keras model
- Create an RDD from the dataset.
- Initialize an instance of elephas.spark model.
- Using spark-submit to submit the script

### CNN: convolution neural networks

**Figure 3:**
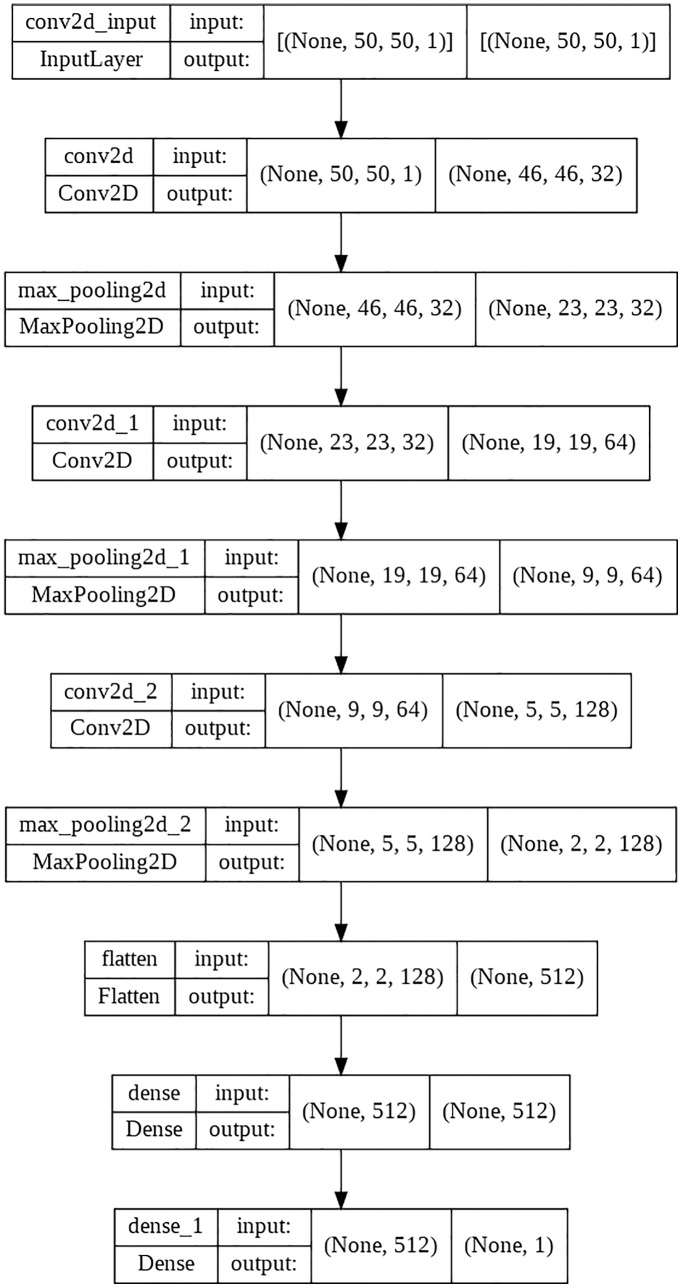
Novel CNN.

CNN is a large FNN inspired by the human visual cortex’s structure (Kang et al., 2014). Because the deep structure of the CNN facilitates hierarchical learning, however, the CNN models must acquire a large quantity of data. Consequently, multiple CNN models are run in clusters rather than on a single computer, as they need significant computing to achieve the required prediction accuracy. CNN generally consists of three layers: convolutional, pooling, and fully connected. The convolutional layer performs a dot product on two matrices, one containing the set of learnable parameters referred to as a kernel and the other containing the limited region of the receptive field. Generates a two-dimensional representation of the picture called an activation map containing information. As with ordinary FCNN, neurons in this layer ultimately connect to all neurons in the previous and following layers. Pooling is a technique for lowering the size of a feature map by employing statistical results such as the mean or maximum to combine the data inside. A basic pooling layer aggregates the correct output of the previous layer’s rectangular single region of neurons. To train deep convolutional networks, an objective function quantifies the error between the network’s output and the desired output. The SGD algorithm is used to solve the optimization problem (Zhang, Choromanska & LeCun, 2015). The novel CNN model used is given in Fig. 3.

### Distributed modelling

Elephas, which is built on the Keras platform, makes use of data-parallel methods using RDDs. Initially, the Keras deep learning models are serialized and distributed across the cluster’s workers, followed by the data and learning parameters. During the training phase, the worker nodes deserialize the model, which is trained on a block of data whose gradients are sent to the driver. The master node’s model is changed *via* an optimizer that receives gradients synchronously or asynchronously. Finally, the distributed processing of the CNN model is done using Algorithm 3. In this, there is a master node that acts as the parameter server model and the worker nodes which have a copy of the model and process in parallel.

**Algorithm 3:** Distributed modelling of proposed model.

At Master Node:
Create a Spark session and Start the Parameter Server
Load Dataset and CNN model
Distribute data to worker nodes
Create a model replica in each worker node
Begin Main
Updated parameter = CALL Worker_node ()
Acquire Write Lock on Master node
Call Worker Node (updated parameters)
Shutdown parameter server and evaluate the master model
End Main
Procedure Worker_Node ()
Broadcast parameters
Start model training
Return updated parameters
End worker node
At Worker Nodes:
Load the dataset distributed from the master node
Load the CNN model replica
Receive parameters from the master node
Start training with the received parameter
Update the parameters and send them to a master node after training

### Result and analysis

The CNN model was deployed using Keras and Elephas libraries in a distributed manner with Apache Spark’s ability. In this work, an epoch is set as 30. Categorical cross entropy was used as a loss function, and the SGD technique with learning parameter 0.1 Elephas leverages data-parallel algorithms employing RDDs. Firstly, the fully convolutional Keras model is serialized and transmitted to workers of the cluster accompanied by data and learning parameters. In the training phase, the worker nodes deserialize the model trained on a data block whose gradients are returned to the controller node. The model at the master node is updated using an optimizer that receives gradients synchronously or asynchronously. The model was executed using normal execution and a parallel distributed model using elephas in synchronous and asynchronous modes. The model parameters are given in Table 1.

**Table 1:** Model parameters.

Parameters	Data
Input data size	50, 50, 1
Model batch size	16,32
Learning rate	0.1
Epochs	30
Optimization	SGD
Loss	Categorical cross-entropy
Training dataset	3,542
Testing dataset	393
No of classes	2
Steps per epoch	221
Spark specification	Version–v3.1.3 Master–local [8] App name–Elephas

**Figure 4:**
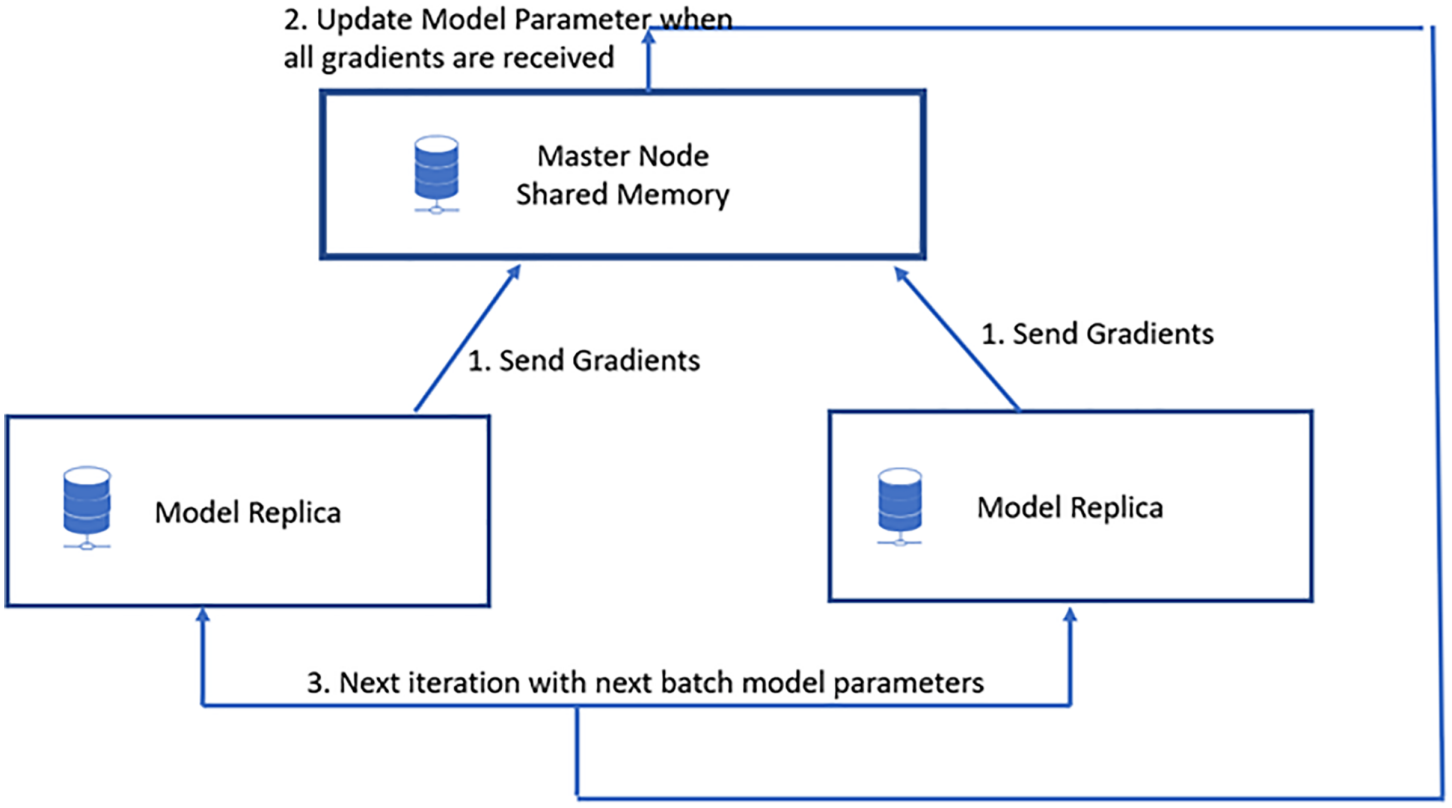
Synchronous data parallel model.

**Figure 5:**
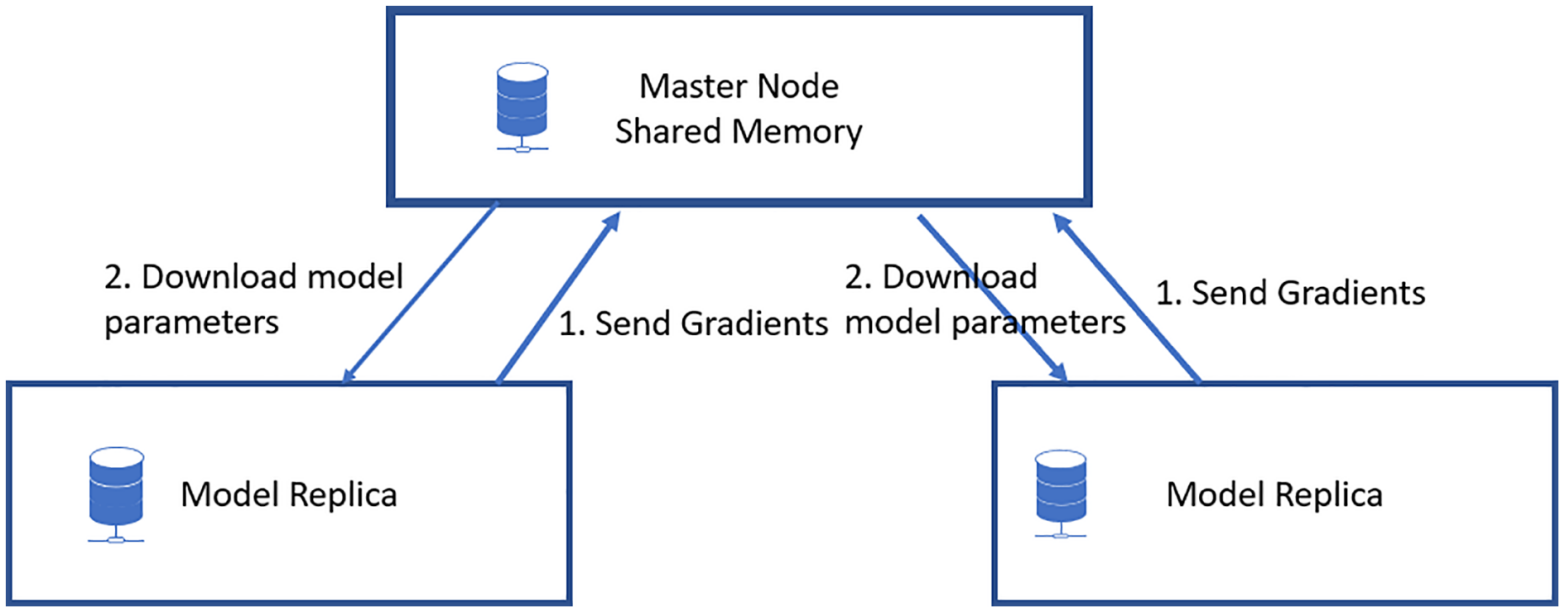
Asynchronous data parallel model.

In data parallelization, the same model is used for all devices, but the model is trained on distinct training instances in each device. Each machine will calculate the discrepancies among its predictions for the training set and label outputs individually. Because each device learns on a separate set of samples, it computes a different set of model updates. However, the method relies on combining the data of all processors for each successive iteration, just as it would on a computer node. As a result, each device must communicate its modifications to all the models on all the other devices. Stochastic gradient descent (SGD) is an iterative technique for determining optimum values. It entails numerous training rounds, with each round’s outcomes being integrated into the models in anticipation of another phase. Synchronous training involves training each device’s local model using various information segments from a data mini-batch. They then send to all devices their locally determined gradients. The model is updated only once all devices have correctly calculated and sent their gradients. After updating the model, it will be sent to all nodes with splits from the next mini-batch. That is, devices are trained on non-overlapping mini-batch splits. Training may come to a halt if a straggler is present. Asynchronous training requires no device to wait for model updates from another device. Instead, the devices may operate autonomously and exchange their findings as peers, or they can interact through one or even more central servers referred to as parameter servers. Each device in the peer architecture executes a loop that collects input, computes gradients, communicates them to all other devices (directly or indirectly), and changes the model to the newest version. These servers aggregate and collect gradients. Synchronous training involves the parameter server computing the most recent version of the model and returning it to the devices, as shown in Fig. 4. Asynchronous training sends gradients to devices that calculate the new model locally. The cycle is repeated in both designs until training is complete, as shown in Fig. 5.

**Figure 6:**
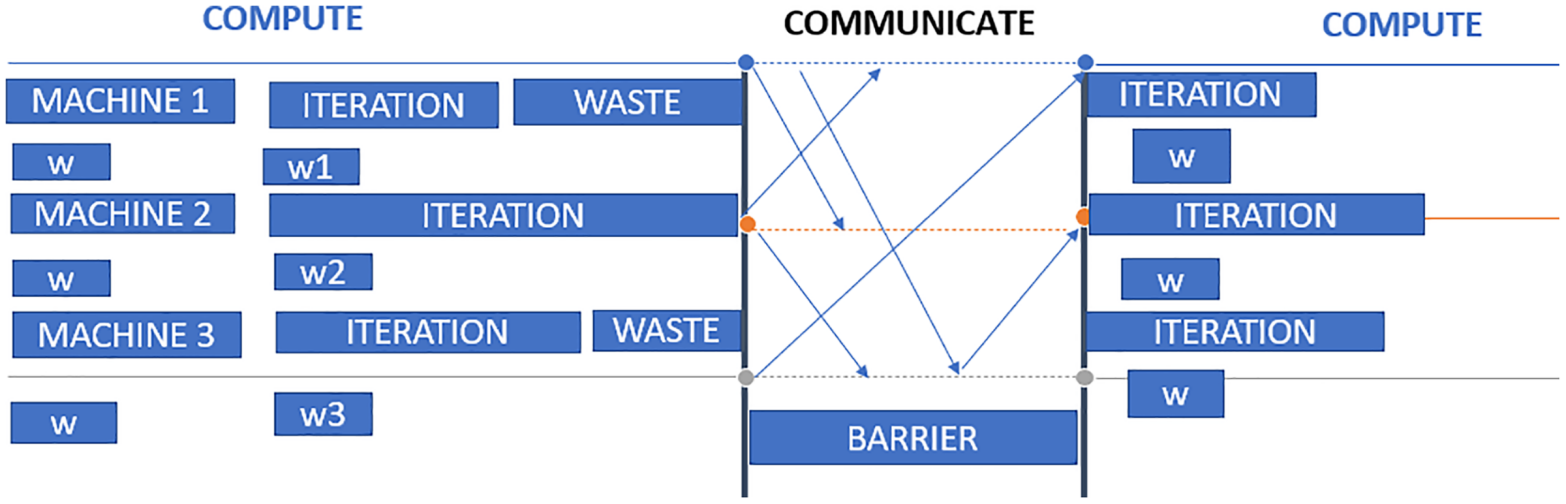
Synchronous data parallel model data transfer.

In Fig. 6, three worker machines get the most recent global weight W from the parameter server and update the local weights w1, w2, and w3. Machine 2 is still working, while machines 1 and 3 have completed their computations. The parameter server can begin aggregating and computing global weight once it has received updated weights from all nodes. Waiting time on worker computers is the primary disadvantage of synchronous data parallelism.

Workers may get global weights from the parameter server as soon as it completes an iteration without waiting for all other workers to complete their iterations. There are many benefits to asynchronous computing. First, we are maximizing the computational power of worker machines with minimal waiting time; second, the partly updated and somewhat out-of-date weight may increase the randomness of training.

However, the asynchronous pattern may also result in slow convergence and poor modeling stability owing to out-of-date parameters. Specifically, the operating iterations for each Computer vary greatly. In reality, system efficiency and algorithm completion must be balanced.

**Figure 7:**
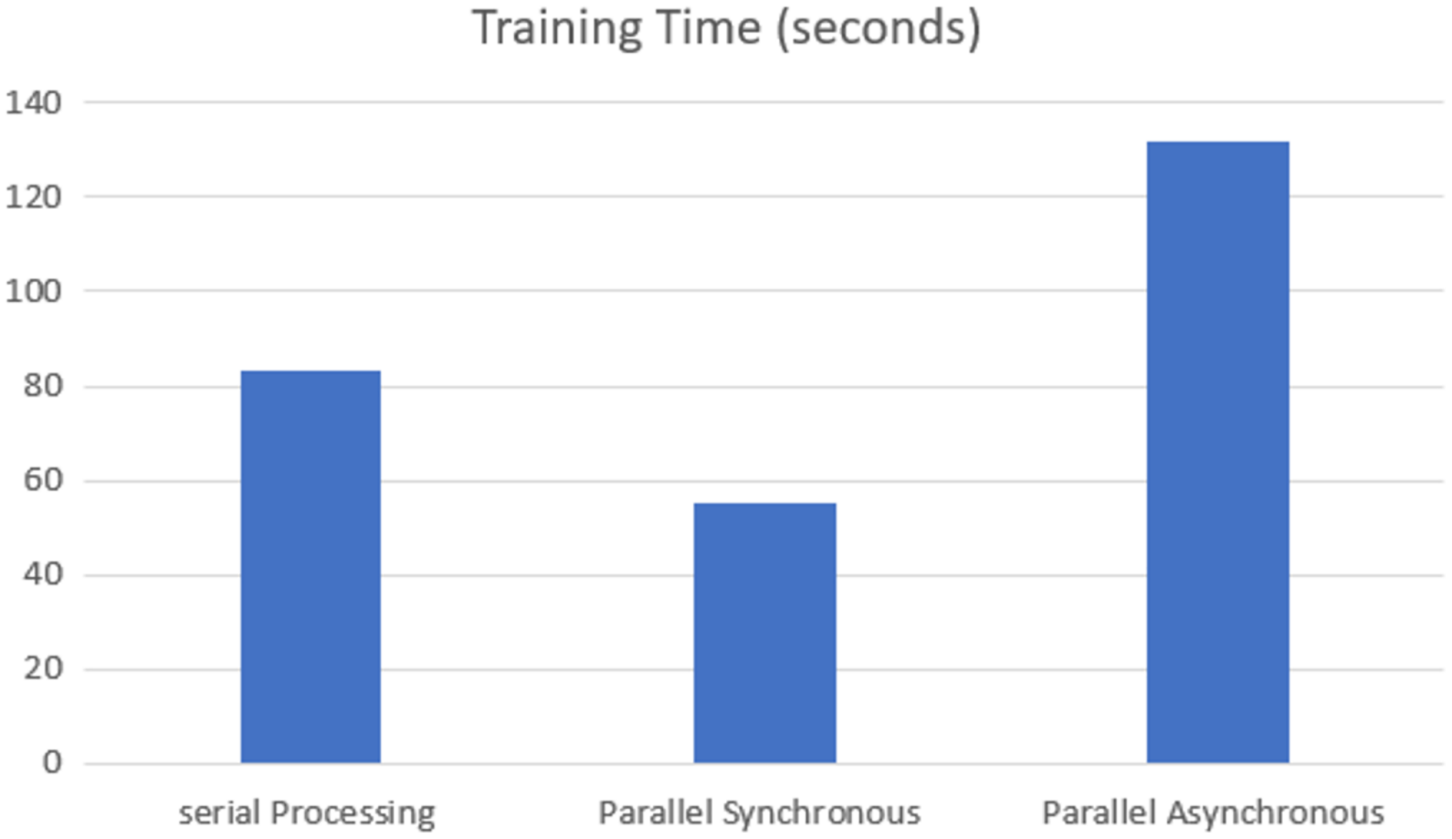
GPU-based training time.

The training time of each model was analyzed on the GPU execution, showing that parallel synchronous execution is faster than serial execution. In the synchronous parallel execution, the training time is reduced by converting the data set into the RDD form and running on the spark model. The three execution modes gave the same accuracy of 97.770% for batch size 16 and the training time, as shown in Fig. 7.

**Figure 8:**
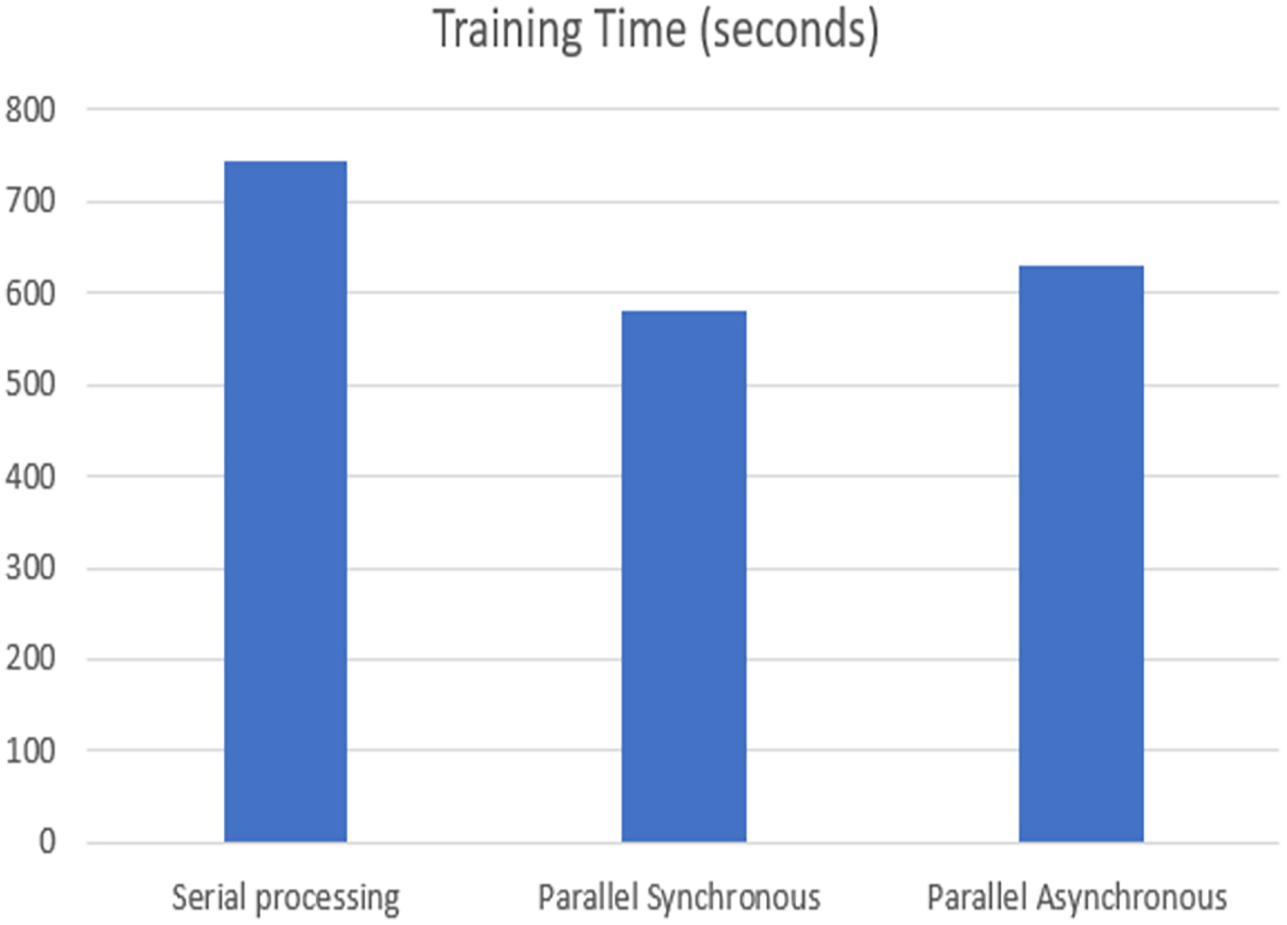
TPU-based training time.

**Figure 9:**
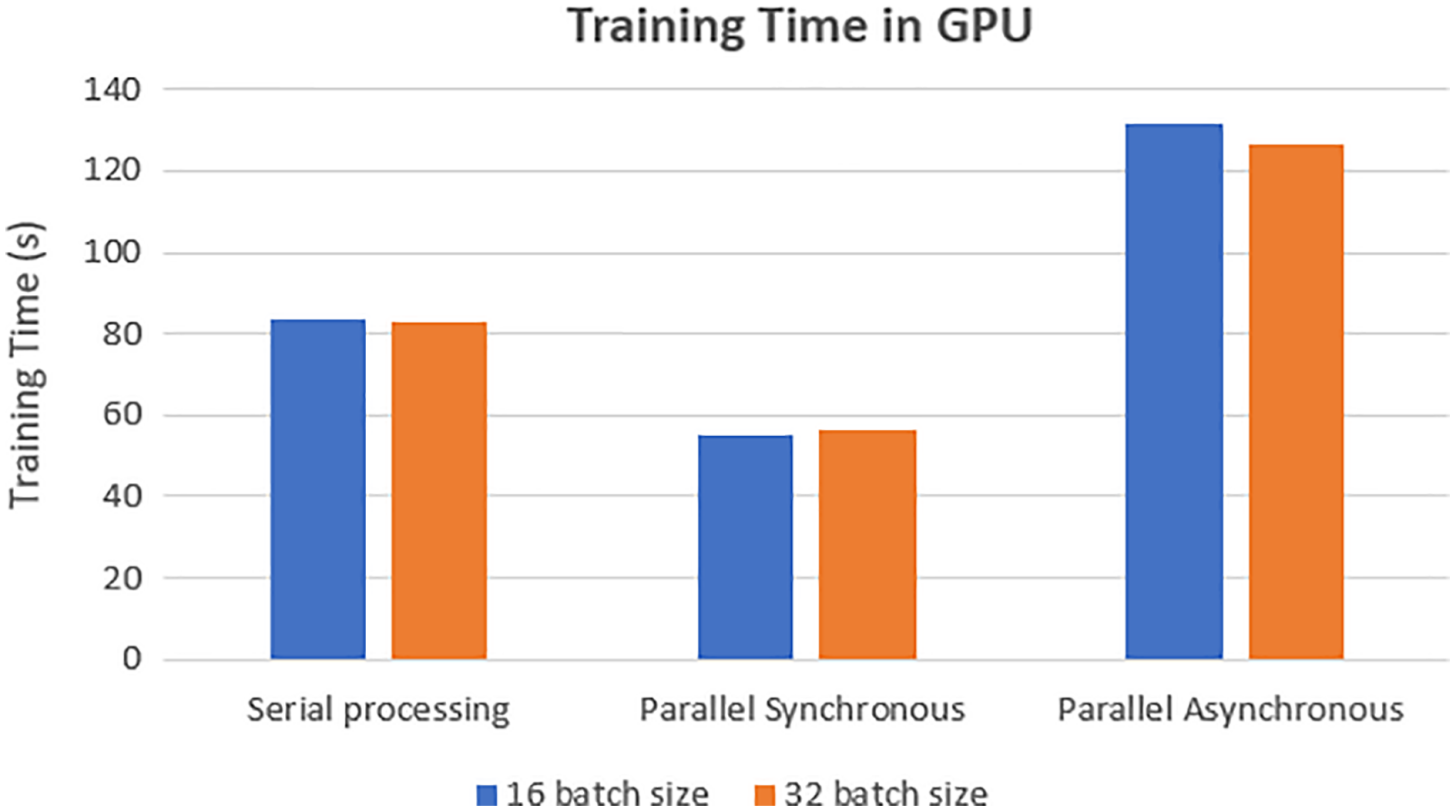
GPU-based training time for batch sizes 16 and 32.

**Figure 10:**
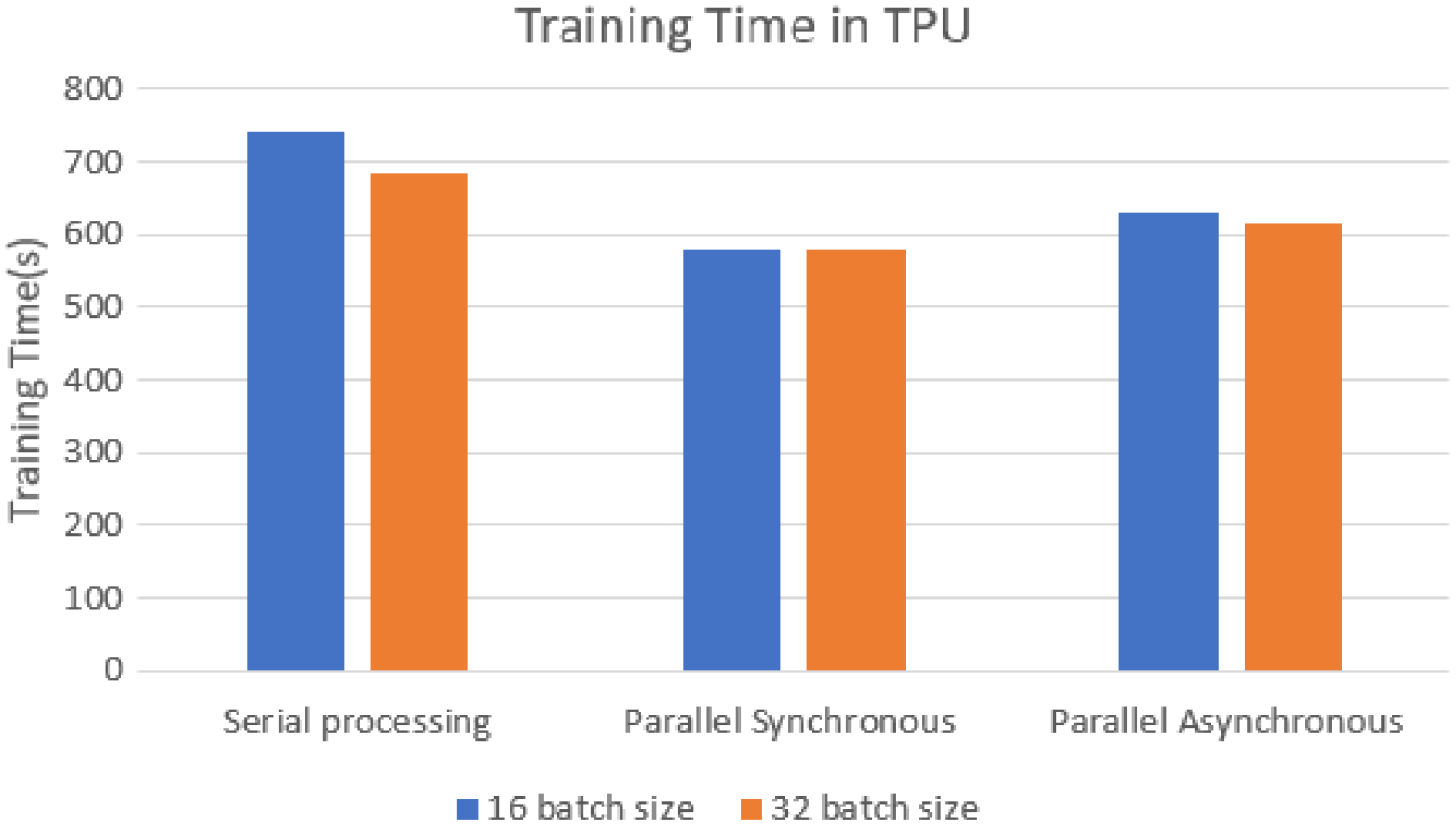
TPU-based training time for batch sizes 16 and 32.

The training time of each model was analyzed on the TPU execution, showing that parallel synchronous and asynchronous execution is faster than serial execution. In the synchronous parallel execution, the training time is reduced by converting the data set into the RDD form and running on the spark model. The three modes of execution gave the same accuracy of 98.72% for batch size 16 and the training time, as shown in Fig. 8. The work was repeated for different batch sizes 16 and 32, and the detailed analysis for GPU and TPU is shown in Figs. 9 and 10.

The speed-up is calculated for the parallel synchronous and asynchronous execution in GPU using Eq. (1) and as shown in Table 2.

(1) $$Speed\; up\; = \; time\; taken\; for\; serial\; execution/time\; taken\; in\; parallel\; execution$$

**Table 2:** Speed up.

Execution method	Speed up
Synchronous parallel in GPU	1.5
Asynchronous parallel in GPU	0.6
Synchronous parallel in TPU	1.28
Asynchronous parallel in TPU	1.17

The speed-up obtained in parallel asynchronous execution is less due to the presence of straggler nodes.

The proposed model is compared with the current state of art models given in Table 3. The existing models with the parallel technique are compared with the proposed model, and it is found the model has better accuracy and is faster. In Ibrokhimov & Kang (2022), transfer learning in VGG19 and Resnet 50 is used along with parallelism to attain an accuracy of 96.9% on the Nvidia Titan X Pascal 12 GB GPU. The average classification accuracy was the only metric used in this study. The work should have discussed the speed-up or resource utilization part. In Moujahid et al. (2020) used transfer learning and parallelism to attain an accuracy of 96.81%.

**Table 3:** Proposed model compared with the existing methods.

Model	Accuracy	Remarks	Reference
VGG16 based	96.81%	Transfer learning parallelism employed	Moujahid et al. (2020)
VGG19 based	96.58%	Transfer learning parallelism employed	Moujahid et al. (2020)
NasNet mobile based	83.37%	Transfer learning parallelism employed	Moujahid et al. (2020)
ResNet152V2 based	96.35%	Transfer learning parallelism employed	Moujahid et al. (2020)
Inception, ResNetV2	94.87%	Transfer learning parallelism employed	Moujahid et al. (2020)
VGG19 and ResNet50	96.6%	Transfer learning parallelism employed	Ibrokhimov & Kang (2022)
Novel CNN model	98.72%	Novel CNN model designed for the specific problem Data parallel model	Proposed model

## Discussion

While doing this work, the primary assumption is that there are no straggler nodes in the experimental setup. With high loads, the system regularly suffers transitory congestion incidents, which causes parameter updates among workers to be delayed. As a result, training convergence degrades as worker nodes behind congested connections struggle to adjust model parameters promptly, postponing all workers. Stragglers lead to prolonged waiting and can cause deadlock in the system (Ravikumar, 2021).

The main limitation is the lack of medical data, which is the primary cause that limits the model’s scalability. Another problem is that there need to be benchmark results for Pneumonia detection.

The model was executed in GPU and TPU for all three modes, and a detailed analysis was done. The key findings are

- Parallel synchronous: At each time step in the synchronous scenario, all copies average their gradients (minibatch). This approach to parallelism places a premium on HPC and the underlying hardware. There is no stale gradient problem in this, but stragglers will occur. Here there is no need for a small step size which was needed in the serial execution of the SGD algorithm. There is low prevention of machine failure. The straggler problem can be mitigated by neglecting the slow worker nodes, using backup workers, *etc*. In synchronous SGD, the larger batch sizes perform better and utilize the best parallelism techniques. The synchronous model works better and is simple. The learning rates can be increased for faster training without compromising performance.
- Parallel asynchronous: The benefit of asynchronous training is that copies may work independently of one another without waiting for others to complete calculating their gradients. This is also where the difficulty lies; There is no assurance that while one duplicate is calculating the gradients for a set of parameters, another still needs to modify the global variables. The global parameters would be updated using stale gradients and calculated using obsolete parameter versions. There is a case of occurrence of a stale gradient problem. The asynchronous execution can be more effective by slowly increasing the worker nodes in the initial epochs. It works well if low learning rates are used in the initial epochs.
- The model can be used for practical real-time analysis of X-ray images at distributed edge devices. Because of the confidential nature of the data, however, this might raise significant privacy issues. Thus patients would just be unwilling to participate. The proposed distributed framework uses distributed deep learning architecture that protects privacy using local differential security and knowledge extraction. Data centralization is optional due to distributed training from federated sources. Distributed methods iteratively study different databases, exchanging research issues and replies across databases instead of sharing the data. In those other terms, one may learn from independent and segregated datasets with patient data never leaving specific clinical institutions. Distributed learning has the potential to ease the use of large amounts of medical data, especially for multinational consortiums.

## Conclusions and Future Work

The findings indicate that the suggested data parallel deep learning model may be utilized to aid healthcare practitioners in identifying pneumonia patients. Even though the suggested approach for diagnosing pneumonia using X-ray images has shown excellent performance, there remains an opportunity for further development. In this model, Spark, an efficient tool for distributed training of the CNN model, is employed due to its support of the wide variety of processing methods with the fault-tolerant way of data sharing among the distributed iterations during the athematic operations. This work’s execution was done in the GPU and TPU platforms for all three execution modes. Utilizing distributed deep learning capabilities may not be as complex as it may seem and may result in a significant performance boost. We obtained an average of 98.72% classification accuracy, which is higher when compared to the existing state of art models. The speed-up varying from 1.2 to 1.5 was obtained in the synchronous and asynchronous parallel model. The speed-up is reduced in the parallel asynchronous model due to the presence of straggler nodes. The straggler mitigation strategies must be developed to obtain the ideal speed up.

In the future, the ensemble model using existing pre-trained algorithms can be distributed to obtain better results. The model can be scaled to the cloud for multiple nodes and can be used for exa-computing in the future. Moreover, this study uses parallel computing to accelerate the training process by distributing data amongst worker nodes. To further speed training in the future, combined data-distributed and model-distributed computation technologies should be employed. New specialized network topologies (explicitly built for X-ray pictures) should be investigated in the future.

## Additional Information and Declarations

### Data Availability

The following information was supplied regarding data availability: The code is available in Zenodo: Aswathy. (2022). aswathyravi2290/Elephas: Elephas for Xray detection (v1.0.0). Zenodo. https://doi.org/10.5281/zenodo.7466309. The data is available at Kaggle: https://www.kaggle.com/datasets/paultimothymooney/chest-xray-pneumonia.
